# Surgical management of 2- or 3-part proximal humeral fractures: comparison of plate, nail and *K*-wires

**DOI:** 10.1007/s12306-020-00686-4

**Published:** 2020-11-30

**Authors:** N. Setaro, M. Rotini, P. Luciani, G. Facco, A. Gigante

**Affiliations:** Clinical Orthopaedics, Department of Clinical and Molecular Sciences, Università Politecnica delle Marche, Via Tronto, 10/A, 60126 Ancona, Italy

**Keywords:** Proximal humeral fractures, Plate, ORIF, Nail, *K*-wire

## Abstract

**Background:**

Proximal humeral fractures (PHFs) are fairly common injuries, and their treatment is a challenge. The aim of this study is to compare clinical and functional outcomes of different osteosynthesis techniques.

**Materials and methods:**

We retrospectively reviewed patients’ files and the hospital’s digital database between March 2002 and April 2018. We treated surgically 148 patients with 2- and 3-part PHFs: 64 with plate and screws, 53 with intramedullary nailing and 31 with retrograde *K*-wires. We constituted three groups according to the type of treatment and two subgroups for each according to the number of fragments (Neer II or Neer III). Disabilities of the Arm, Shoulder and Hand (DASH) and Short Form-12 (SF-12) scores were recorded.

**Results:**

Mean DASH and SF-12 scores both from the group treated with plate (Group I) and the one subjected to intramedullary nailing (Group II) were statistically superior to results from the patients treated by retrograde *K*-wires (Group III), while nails showed better functional results than the locking plates. In the first two groups, no difference was found between Neer II and III subgroups, while in Group III the DASH scores were significantly better in Neer II subgroup than those in Neer III subgroup. Avascular necrosis was the most frequent cause of revision surgery in Group I (4 cases) where we had 8 cases of reintervention (12.5%). In Group II, the subacromial impingement was the only cause for revision surgery with 3 cases (5.6%).

**Conclusions:**

Intramedullary nails showed better functional results and a lower complication rate than the locking plates. Both techniques showed superior results compared to those available with retrograde *K*-wires. So the nail seems to be a more reliable and adequate method for treating 2- and 3-part proximal humeral fractures.

## Introduction

Fractures of the proximal end of the humerus in the adult–elderly patient are fairly common injuries.

Their incidence is 4% of all adult fractures and rank third among osteoporosis fractures following those of the femur and wrist. Most patients with a proximal humerus fracture are females and over 60, with a 3:1 ratio compared to males [1–3]. The wide variety of morphologies and fracture treatment options, ranging from conservative treatment to various osteosynthesis methods up to arthroplasty, make studies on the treatment of proximal humeral fractures difficult to perform. The conclusion of a recent Cochrane review was that no evidence-based recommendation on the treatment of proximal humerus fractures can be derived from currently available data [4].

The most appropriate treatment should therefore be chosen taking into account the biological age and bone quality of the specific patient as well as his/her functional requests, comorbidities, compliance and personal wishes.

ORIF offers a reconstruction that respects the shoulder anatomy, but it presents several complications like avascular necrosis of the humeral head, screws cut-out, loss of reduction, nonunion and impingement syndrome [5].

Intramedullary nailing is a less invasive surgical procedure with a closed fixation that allows a rapid functional rehabilitation. However, it presents a minor stability and a high technical difficulty in fractures with more than 2 fragments. Moreover, it can cause a lesion of the rotator cuff and subacromial impingement due to the protrusion of the nail [6].

The percutaneous technique with Kirschner wires shows advantages such as the maintenance of the fracture hematoma, the preservation of the vascular system, the shortest duration of the operation; however, this technique also shows a lower stability, a longer immobilization period and the danger of migration of the wires [7].

The aim of this study was to retrospectively evaluate the clinical and functional outcomes of patients with two and three fragments fractures of the proximal humerus treated with different osteosynthesis techniques at our hospital over the last 16 years.

## Materials and methods

The retrospective evaluation included all patients coming to our attention for a proximal humerus fracture type II or III according to Neer classification and surgically treated in Clinical Orthopaedics, Ancona between March 2002 and April 2018.

All the patients included in the study were treated by the same equipe; all fractures were classified according to Neer’s criteria. The choice of treatment was based on the metaphyseal bone stock, the possibility of obtaining a stable closed reduction and the state of medial arch. Patients with concomitant fractures, those with a previous fracture of the proximal humerus to the same side, those who were no longer reachable by telephone or who had died have been excluded.

At the time of the trauma, all patients were clinically evaluated and underwent X-Ray in a true glenoid antero-posterior projection and lateral scapular projection. CT-scan was reserved for patients with displaced fractures to better evaluate the number, size and position of the fragments. Thanks to the radiographic image database of our center, it was possible to review the pre- and postoperative radiographs and the control radiographs with fracture healing at follow-up.

At June 2019, patients’ files and the hospital’s digital database were reviewed retrospectively. In the selected time period of the 206 patients with 2- and 3-fragment fractures of the proximal humerus, 168 (101 Neer II and 67 Neer III) underwent surgery. Once all candidates had been contacted, 148 patients were available and met the inclusion criteria for this study. Of these, 64 (37 Neer II and 27 Neer III) were treated by osteosynthesis with plate and screws, 53 (34 Neer II and 19 Neer III) underwent intramedullary nailing while 31 (20 Neer II and 11 Neer III) were treated with retrograde K (Kirschner) wires. Subsequently, we constituted three groups according to the type of treatment and two subgroups for each treatment according to the number of fragments (Neer II or Neer III).

We filled in a demographic form for each patient, in which we reported: date of birth, gender, weight, height, date of trauma, age at surgery time, total months of follow-up, injured side. All patients were subjected to DASH (Disability of the Arm, Shoulder and Hand) score and SF-12 (Short Form-12) score by telephone interview.

### Surgical technique

The surgical technique for patients treated with plate and screws fixation included a deltoid-pectoral access, a temporary reduction in the fracture fragments by using *K*-wires and finally positioning of a Philos plate (Synthes) placed 5-7 mm below the great tuberosity to reduce the risk of subacromial impingement. The plate was then locked with at least 4 proximal screws and, in case of poor bone quality, all proximal screws were used. We were also really careful about positioning inferior screws in order to give a medial support to the surgical humeral neck. Distally we placed 3 diaphyseal screws, one of which was used for compression of the plate on the bone and 2 more for angular stability.

The surgical technique for patients treated with intramedullary nail involved close reduction in fracture fragments with temporary fixation using percutaneous *K*-wires to hold the fragments in place, if needed. A small antero-lateral incision was then made on the cutaneous projection of the greater tuberosity, whose apex was used as an access route for anterograde nailing. In case of fracture of the greater tuberosity, entry point was slightly more medial. A guide wire for the reamer was introduced under C-arm control. The reaming guide was then used to introduce a Trigen nail (Smith & Nephew). Nail interlocking was obtained by 2 or more proximal screws and 2 distal screws.

In patients treated with *K*-wires, the surgical technique involved closed reduction in the fracture fragments and stabilization with 3 retrograde percutaneous *K*-wires inserted under fluoroscopic control. *K*-wires were removed 30 days after surgery.

The rehabilitation program, in patient treated with plate and nail, required a sling immobilization of the limb for 2 weeks. Passive shoulder exercises without gravity and active elbow movements were allowed on the very first postoperative day. After 2 weeks, progression to active assisted motion was started avoiding internal and external rotation until 40 days after surgery. Patients treated with *K*-wires required a sling immobilization of the limb for 4 weeks and were allowed only active elbow movements during this time. After 30 days, passive and active shoulder exercises were started avoiding internal and external rotation until 40 days after surgery.

### Statistical analysis

The categorical variables were expressed in numbers and percentages. The continuous variables were expressed as means and standard deviation (DS). Data from three groups were compared using the Mann–Whitney test, the t test and Fisher’s exact test when it was appropriate. Statistical analyses were carried out using SPSS (version 21.0; IBM, Armonk, NY, USA). A *p* < 0.05 was considered significant.

## Results

The average age at the time of trauma of patients treated with plate and screws was 61.5 ± 25 years; the mean follow-up was 48 ± 16.1 months; dominant limb was affected in 42 cases. In the group treated with intramedullary nail, dominant limb was affected in 37 cases, the mean age at trauma was 64 ± 21.2 years, and the mean follow-up was 40.4 ± 15.4 months. Finally, the mean age at trauma of patients treated with K wires was 67.9 ± 15.7 years; the mean follow-up was 45 ± 25.4 months; dominant limb was affected in 21 cases. We did not find statistically significant differences between the three groups about demographic data.

Tables 1 and 2 show the mean DASH and SF-12 scores of each approach. Mean DASH and SF-12 scores both from the group treated with plating and the one subjected to intramedullary nailing were statistically superior to results from the patients treated by retrograde *K*-wires (*p* < 0.05), while nails showed better functional results than the locking plates (*p* < 0.05). There are no differences between Neer II fractures and Neer III fractures in both plate and nail. Instead, patients treated with retrograde *K*-wires showed a significantly higher DASH score in Neer III fractures than in Neer II fractures (*p* < 0.05).

**Table 1:** Mean DASH and SF-12 scores in plate and screws (Group I), nail (Group II) and *K-*wires (Group III)

	Group I	Group II	Group III
DASH	27.1 (8.2)	14.3 (6.2)	45.7 (12.2)
SF-12	47.3 (2.4)	54.7 (2.6)	39.7 (2.5)

DASH, disabilities of the arm, shoulder and hand; SF-12, Short Form-12

Data are presented as mean (standard deviation)

**Table 2:** Mean DASH and SF-12 scores in plate and screws (Group I), nail (Group II) and *K*-wires (Group III) according to the number of fragments (Neer II or Neer III)

	Group I		Group II		Group III	
	Neer II	Neer III	Neer II	Neer III	Neer II	Neer III
DASH	25.6 (7.5)	29.3 (9.3)	12.5 (5.1)	17.3 (7.2)	40.2 (10.5)	53.4 (14.8)
SF-12	47.9 (2.3)	46.7 (2.6)	55.6 (2.6)	53.8 (2.7)	42.7 (2.5)	36.7 (2.6)

DASH, disabilities of the arm, shoulder and hand; SF-12, Short Form-12

Data are presented as mean (standard deviation)

Among the 64 patients treated with plate and screws, 11 complained of pain from fixation devices in the months following surgery and 8 of these underwent a subsequent surgical revision (12.5%). Although the maximum number of screws was used in 2 cases, there was a varus dislocation and a second reduction and fixation with plate was required. In 2 patients, impingement occurred and the plates were removed after fracture healed; in both cases the plate was positioned 5 mm below the great tuberosity. Finally, 4 patients with avascular necrosis required a revision with reverse prosthesis. Out of the 53 patients treated with intramedullary nailing, 3 reported subacromial impingement due to nail protrusion and required a second intervention to remove the nail (5.6%). Finally, patients treated with retrograde *K*-wires had two superficial infections of wires that were treated with 5 days of oral antibiotics obtaining infection resolution.

## Discussion

The best treatment for 2- and 3-part dislocated fractures in elderly patients remains under doubt. Displaced proximal humerus fractures commonly receive surgical treatment with the aim to achieve satisfactory results, despite controversies concerning the advantages over conservative treatment [8].

Osteosynthesis by locking plate is the most common surgical treatment for these fractures [4, 8], but it is associated with a high rate of complications [9–11]. Intramedullary nailing becomes an attractive alternative for proximal humerus fractures due to superior biomechanical advantages, including greater rigidity for valgus, extension and torsion stresses [12].

Several studies have compared nailing and plating for the treatment of displaced proximal humerus fractures, reporting various clinical outcomes and complications with conflicting results [8, 13, 14]. For this reason, the best surgical approach still remains controversial. To our knowledge, there is only one meta-analysis available which compared the clinical results and complications between these two approaches in patients with displaced proximal humeral fractures; this study displayed similar effects of the locking plate and intramedullary nail on the Constant score and the rates of total complication [15].

In the present study, when considering DASH and SF-12 scores, statistically significant difference was found between plating group and the one treated by intramedullary nailing, unlike what was reported in two randomized controlled trials in which the results were similar [16, 17]. Worse DASH and SF-12 scores were observed in plate treated patients compared to nail treated ones. In particular, patients treated with plate had worse scores for activities that required an abduction of the arm over 90°–100°.

However, both groups showed better mean DASH and SF-12 scores than the group treated by retrograde *K*-wires, resulting in a statistically significant difference. Our study confirmed the results reported by Edelmann et al. in which the *K*-wire group showed a significantly worse functional outcomes than the group treated with angle-stable implants in both Constant and DASH scores of patients with three- and four-fragment fractures of the proximal humerus [18].

No statistically significant differences were observed either in the DASH score or in the SF-12 score between patients with two and three fragment fractures treated with plate and screws or intramedullary nail. Finally, statistically significant difference was observed in the DASH score of patients with two and three fragment fractures treated with retrograde *K*-wire; 3-fragment fractures had worse clinical results probably due to the greater difficulty in reducing and maintaining stability compared to 2-fragment fractures.

Regarding complications, unlike other studies that observed no differences between plating and intramedullary nailing [19], the present study reported a higher rate of surgical revision for patients treated with locking plate (12.5%) compared to patients treated with nail (5.6%).

Avascular necrosis (AVN) depends on the type of fracture, on the metaphyseal extension, on the integrity of the calcar [20]. Furthermore, we must also consider the important damage that the surgeon can cause to the vascular system during ORIF reduction [21]. In our study, we had 4 cases of AVN (6.3%) and it was the most frequent cause that led to a revision of the implant in Group I.

This study has several limitations: (1) it is a retrospective and non-randomized study; (2) some patients lost to follow-up. Further prospective studies are needed to evaluate the best fixation method for the treatment of proximal humerus fractures.

## Conclusions

Although a reliable comparison between groups is not possible due to the non-randomization of the groups, intramedullary nails showed better functional results and a lower complication rate than the locking plates in the treatment of 2- and 3-part proximal humeral fractures. Both techniques showed superior results compared to those available with retrograde *K*-wires. Patients undergoing intramedullary nailing showed less frequently pain/discomfort from fixation devices; in particular, the nail scored better in activities that required arm abduction beyond 90°–100° compared to the plate. Therefore, the nail seems to be a more reliable and adequate method for treating 2- and 3-part proximal humeral fractures than plate and *K*-wires.
